# Prognostic value of CHADS_2_ and CHA_2_DS_2_-VASc scores for post-discharge outcomes in patients with acute coronary syndrome undergoing percutaneous coronary intervention

**DOI:** 10.1097/MD.0000000000021321

**Published:** 2020-07-24

**Authors:** Xiaoteng Ma, Qiaoyu Shao, Lisha Dong, Yujing Cheng, Sai Lv, Hua Shen, Jing Liang, Zhijian Wang, Yujie Zhou

**Affiliations:** Department of Cardiology, 12th Ward, Beijing Anzhen Hospital, Capital Medical University, Beijing Institute of Heart Lung and Blood Vessel Disease, Beijing Key Laboratory of Precision Medicine of Coronary Atherosclerotic Disease, Clinical Center for Coronary Heart Disease, Capital Medical University, Beijing, China.

**Keywords:** acute coronary syndrome, CHA_2_DS_2_-VASc score, CHADS_2_ score, major adverse cardiovascular events, percutaneous coronary intervention

## Abstract

The CHADS_2_ and CHA_2_DS_2_-VASc scores were initially developed to assess the risk of stroke or systemic embolism in patients with atrial fibrillation (AF). Recently, these two scoring systems have been demonstrated to predict long- and short-term cardiovascular (CV) outcomes in many patient cohorts. However, to the best of our knowledge, their prognostic value has not been fully elucidated in patients with acute coronary syndrome (ACS) undergoing percutaneous coronary intervention (PCI). This study aimed to investigate the association of CHADS_2_ and CHA_2_DS_2_-VASc scores with CV outcomes in such patients.

We included a total of 915 ACS patients undergoing PCI in this study. CHADS_2_ and CHA_2_DS_2_-VASc scores were calculated from data collected before discharge. The primary endpoint was defined as a composite of major adverse CV events (MACE) including overall death, nonfatal stroke, nonfatal myocardial infarction (MI) and unplanned repeat revascularization. We assessed MACE's relationship to CHADS_2_ and CHA_2_DS_2_-VASc scores using Cox proportional-hazard regression analyses.

Mean follow-up duration was 918 days. MACE occurred in 167 (18.3%) patients. A higher CHADS_2_ score was associated with reduced event-free survival (EFS) from MACE (logrank test, *P* = .007) with differences potentiated if stratified by CHA_2_DS_2_-VASc score (logrank test, *P* < .001). Univariate analysis showed that both CHADS_2_ and CHA_2_DS_2_-VASc scores were good predictors of MACE. In the multivariate Cox proportional-hazard regression analysis, CHA_2_DS_2_-VASc score (hazard ratio [HR], 1.15; 95% confidence interval [CI] 1.04–1.27; *P* = .007) remained a useful predictor of MACE; however, CHADS_2_ score was no longer associated with increased risk of MACE. C-statistics for CHA_2_DS_2_-VASc score, GRACE (Global Registry of Acute Coronary Events) hospital discharge risk score (GRACE Score) and SYNTAX (Synergy between PCI with TAXUS and Cardiac Surgery) Score II (SS II) in predicting MACE were 0.614, 0.598, and 0.609, respectively.

CHA_2_DS_2_-VASc score was an independent and significant predictor of MACE in ACS patients undergoing PCI, and its discriminatory performance was not inferior to those of GRACE Score and SS II.

## 1 Introduction

Acute coronary syndrome (ACS) patients have a wide spectrum of risks for subsequent cardiovascular (CV) causes of morbidity and mortality.^[1]^ Myocardial revascularization, especially percutaneous coronary intervention (PCI), is deemed one of the most effective therapeutic methods for reducing risk of death and improving the prognosis of ACS patients. However, even when treated with PCI and guideline-directed medical therapy, patients with a definite diagnosis of ACS are still at increased risk of adverse CV events.^[2]^ Accordingly, accurate and early identification of such patients at high CV risk would facilitate better clinical management in the future. Unfortunately, there is still a lack of simple and convenient risk assessment tools for predicting adverse CV events in ACS patients undergoing PCI.

The CHADS_2_ (**C**ardiac failure; **H**ypertension; **A**ge ≥75 years; **D**iabetes; previous **S**troke or transient ischemic attack [TIA] [doubled]) and CHA_2_DS_2_-VASc (**C**ardiac failure; **H**ypertension; **A**ge ≥ 75 years [doubled]; **D**iabetes; previous **S**troke or TIA [doubled]; **V**ascular disease; **A**ge 65–74 years; and **S**ex category) scores were initially developed to assess risk of stroke or systemic embolism and to guide antithrombotic therapy in patients with atrial fibrillation (AF).^[3,4]^ In fact, most of the variables in these 2 scoring systems are also important risk factors of atherosclerotic CV diseases independent of the cardioembolic pathway; therefore, it is assumed that such scoring systems might have important applications in predicting a wider range of pathophysiologically related CV events beyond the conventional scope of AF.^[5]^ Indeed, the CHADS_2_ and CHA_2_DS_2_-VASc scoring systems have proved to be valuable for predicting the incidence of nonfatal ischemic events and death in many patient groups, regardless of the presence or absence of AF.^[5–18]^ Of note, several studies have reported that high CHADS_2_ and CHA_2_DS_2_-VASc scores are associated with a significant increase in long- and short-term adverse CV events in patients with ACS^[6,7,13]^ or in those undergoing PCI^[8,9,11,12,18]^. A previous study found no significant difference between CHADS_2_ and CHA_2_DS_2_-VASc scores in predicting mortality in ACS patients.^[6]^ However, additional components of the CHA_2_DS_2_-VASc score that are not included in the CHADS_2_ score—namely, peripheral artery disease (PAD), myocardial infarction (MI) and female sex—are associated with worse clinical outcomes in ACS patients.^[19,20]^ A recent study demonstrated that the CHA_2_DS_2_-VASc score had better predictive value for clinical outcomes than the CHADS_2_ score.^[7]^ Nevertheless, the usefulness of these scores for predicting adverse events in ACS patients undergoing PCI has not been exclusively and adequately studied.^[16]^

The purpose of this study was twofold:

1. to investigate the predictive utility of the CHADS_2_ and CHA_2_DS_2_-VASc scores for major adverse CV events (MACE) in ACS patients undergoing PCI; and
2. to compare the discriminatory performance of the CHA_2_DS_2_-VASc score with that of the GRACE (Global Registry of Acute Coronary Events) hospital discharge risk score (GRACE Score) and SYNTAX (Synergy Between PCI With TAXUS and Cardiac Surgery) Score II (SS II) for MACE.

## 2 Methods

### 2.1 Study population

We enrolled 998 consecutive patients who were admitted to our CV center, diagnosed with ACS and treated with primary or elective PCI during the period from June 2016 to March 2017 into a prospective registry.^[21]^ ACS was diagnosed according to American College of Cardiology (ACC)/American Heart Association (AHA) guidelines.^[22,23]^ Patients with the following conditions were excluded: death before discharge, prior coronary-artery bypass grafting (CABG), renal dysfunction with creatinine clearance (CrCl) < 15 mL/min or chronic dialysis, Killip class > II, New York Heart Association (NYHA) class III/IV and left ventricular ejection fraction (LVEF) < 30%. Two patients were excluded because of missing follow-up data despite at least 4 separate attempts to contact them. Ultimately, 915 patients were retrospectively identified as eligible and were included in the final analysis.

This study was performed in accordance with the Helsinki Declaration of Human Rights. It was approved by the Ethics Committee of Beijing Anzhen Hospital, Capital Medical University, Beijing, China. The requirement for informed patient consent was waived in view of the retrospective nature of the study.

### 2.2 Measurements

In addition to demographic data, we documented traditional CV risk factors and medication history via a questionnaire. After collection of heparinized plasma samples from the central laboratory of Beijing Anzhen Hospital, we analyzed all laboratory parameters immediately. Cardiac failure was defined as the presence of signs/symptoms of congestive heart failure (CHF), current treatment for CHF or objective evidence of reduced ejection fraction (LVEF < 40%). Patients with vascular diseases related to the aorta and other arteries than the coronary, accompanied by exercise-related intermittent claudication, revascularization surgery, reduced or absent pulsation, angiographic stenosis of ≥50% or combinations of these characteristics, were identified as having PAD. Patients with previous ischemic stroke or transient ischemic attack were defined as having cerebrovascular accident (CVA). Chronic kidney disease (CKD) was defined as CrCl < 60 mL/min, which we calculated using the Cockcroft–Gault formula^[24]^ from the patient's age, weight and serum creatinine (sCr) concentration recorded on admission. The LVEF used was the lowest of the values recorded before the index PCI.

### 2.3 PCI procedure

All patients were given loading doses of aspirin (300 mg) and either clopidogrel (300 mg) or ticagrelor (180 mg) before intervention unless they had already received antiplatelet drugs. Subsequently, all patients were required to take aspirin for lifetime and a P2Y12 inhibitor for at least 1 year after intervention. Coronary angiography and PCI were performed using standard techniques.^[25]^ Treatment strategy, balloon dilatation or stent implantation technique, and choice of a particular balloon or stent were all left to the operator's discretion.

### 2.4 CHADS_2_ and CHA_2_DS_2_-VASc scores

CHADS_2_ and CHA_2_DS_2_-VASc scores were calculated from data collected before discharge according to their respective criteria.^[3,4]^ We calculated CHADS_2_ score by assigning 1 point each for cardiac failure, hypertension, age ≥75 years and diabetes; and 2 points for previous CVA. We calculated CHA_2_DS_2_-VASc score by assigning 1 point each for cardiac failure, hypertension, diabetes, vascular disease (including prior/current MI, PAD or aortic plaque), age 65 to 74 years and female sex; and 2 points each for age ≥75 years and previous CVA. In view of the relationship between these 2 scoring systems and adjusted stroke rate reported in previous studies,^[3,26]^ we divided these 2 scores into low, intermediate and high categories, respectively, as follows: CHADS_2_ scores 0, 1–2, and ≥3; CHA_2_DS_2_-VASc scores ≤1, 2–4, and ≥5.

### 2.5 GRACE score

We calculated GRACE Score (http://www.outcomes-umassmed.org/grace/), which estimates risk of death or MI within the 6 months following hospital discharge, from a patients’

1. medical history (age, history of CHF and history of MI);
2. findings at hospital presentation (resting heart rate, systolic blood pressure [SBP] and ST depression in electrocardiogram [ECG]); and
3. findings during hospitalization (initial sCr level, elevated cardiac enzymes and in-hospital PCI).

### 2.6 SYNTAX score II

The SS II has been described in full previously.^[27]^ Briefly, it consists of 2 anatomical variables (SYNTAX Score and left-main disease) and 6 demographic and clinical factors (age, CrCl, LVEF, sex, chronic obstructive pulmonary disease [COPD], and PAD). Details are available on the SYNTAX Score website (www.syntaxscore.com).

### 2.7 Follow-up and end points

All patients were followed up at 1 month and then every 6 months after hospital discharge. Trained personnel who were blinded to risk scores obtained information on adverse events via telephone contact with patients or their family members using a standardized questionnaire; the adverse events were then ascertained from a careful review of corresponding medical records. The primary endpoint of the study was incidence of MACE, defined as overall death, nonfatal stroke, nonfatal MI or unplanned repeat revascularization. Death was considered to be of CV cause in origin unless a definite non-CV cause could be identified. Stroke was defined as ischemic cerebral infarction (ICI) with evidence of neurological dysfunction requiring hospitalization and with clinically documented lesions on the brain as shown by computed tomography (CT) or magnetic resonance imaging (MRI). MI was defined as elevated levels of cardiac enzymes, such as cardiac troponin or the MB fraction of creatine kinase, exceeding the upper limit of the normal range with either ischemic symptoms or ECG changes implicating ischemia. The presence of new pathological Q waves in ≥2 contiguous ECG leads was also diagnosed as MI. Within 1 week after the index PCI, only Q-wave MI was adjudicated as MI. Unplanned repeat revascularization was defined as any nonstaged revascularization after the index PCI. Staged revascularization was defined as scheduled revascularization within 90 days after the index PCI without retreatment of a coronary-artery territory that had been treated during the index PCI; or a revascularization status of emergency, urgency or salvage. The most severe endpoint was selected for primary-endpoint analysis if >1 MACE endpoint occurred during follow-up (death > stroke > MI > revascularization). If >1 stroke or MI or revascularization occurred, the first stroke, MI or revascularization was selected.

### 2.8 Statistical analysis

CHADS_2_ and CHA_2_DS_2_-VASc scores were analyzed in 2 ways:

1. as continuous variables; and
2. as ordinal variables (low vs intermediate vs high).

Continuous variables with parametric distributions are reported as the mean ± standard deviation (SD) and those with nonparametric distributions are reported as median and interquartile ranges. Categorical variables are reported as numbers and percentages. We analyzed differences in 2 continuous variables using Student's *t* test or the Mann–Whitney *U* test. Differences in categorical variables were tested using the chi-square test or Fisher's exact test, as appropriate. We constructed survival curves as stratified by baseline CHADS_2_ and CHA_2_DS_2_-VASc scores using the Kaplan–Meier procedure with Mantel–Haenszel logrank testing for significance. Hazard ratios (HRs) with 95% confidence intervals (CIs) were calculated using Cox proportional-hazard regression analyses. Predictors of MACE identified via univariate analysis were tested in the multivariate analysis. We selected variables with a univariable significance level of ≤.10 and excluded those that would have caused internal correlations. Female sex, age, hypertension, diabetes, previous CVA, previous MI, current diagnosis of MI, PAD, and cardiac failure are components of the CHADS_2_ and CHA_2_DS_2_-VASc scores, and we believed these variables would cause internal correlations with the variables of the CHADS_2_ and CHA_2_DS_2_-VASc scores. The validity of the proportionality assumption was verified for all covariates by a visual examination of the log (minus log) curves and a test based on Schoenfeld residuals. The discriminatory performances of the CHA_2_DS_2_-VASc score, GRACE Score and SS II for MACE were all assessed using C-statistics. We compared the discriminatory performance of the CHA_2_DS_2_-VASc score with those of GRACE Score and SS II via DeLong's method. Analyses were performed using SPSS version 24.0 (IBM Corp., Armonk, NY) and R software version 3.5.3 (R Foundation for Statistical Computing, Beijing, China). A 2-sided *P*-value < .05 was considered significant.

## 3 Results

**Figure 1:**
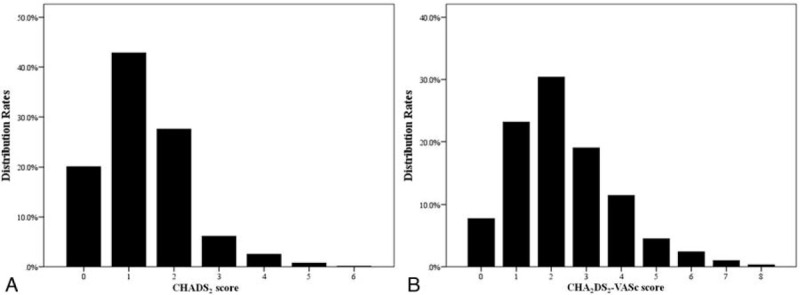
(A) Distribution of CHADS_2_ scores over total number of patients. (B) Distribution of CHA_2_DS_2_-VASc scores over total number of patients.

A total of 915 patients with ACS undergoing PCI were included in the present study. These patients had a mean age of 60 ± 10 years; 23.4% were females. CHADS_2_ and CHA_2_DS_2_-VASc scores of total patients are shown in Figure 1A and B, respectively.

Mean follow-up duration was 918 days. Of the total number of patients, 167 (18.3%) developed MACE, which included 21 (2.3%) deaths, 28 (3.1%) events of nonfatal MI, 148 (16.2%) cases of unplanned repeat revascularization and 11 (1.2%) nonfatal strokes. Of these 167 patients, 17 (1.9%) suffered two, 7 patients (0.8%) suffered three and 2 (0.2%) patients suffered four MACE events.

Patients’ baseline demographic and clinical characteristics are presented in Table 1, stratified by development of MACE. Compared with those without MACE, patients with MACE had higher rates of cardiac failure (12.6 vs 3.9%; *P* < .001), diabetes (52.7% vs 38.9%; *P* = .001), previous MI (25.7% vs 18.6%; *P* = .036), past PCI (26.3% vs 18.4%; *P* = .021) and PAD (22.8% vs 6.6%; *P* < .001). Those with MACE also had higher levels of fasting plasma glucose (FPG; *P* < .001) and glycated hemoglobin A1c (HbA1c; *P* < .001). LVEF was significantly lower in patients with MACE (*P* = .029). Use of medications did not differ between patients with and without MACE at discharge. Angiographic findings and interventional characteristics of patients are presented in Table 2, stratified by development of MACE. Compared with those without MACE, patients with MACE had higher rates of multivessel or left-main disease, restenotic lesions, and lesions > 20 mm long (all *P* ≤ .006).

**Table 1:**
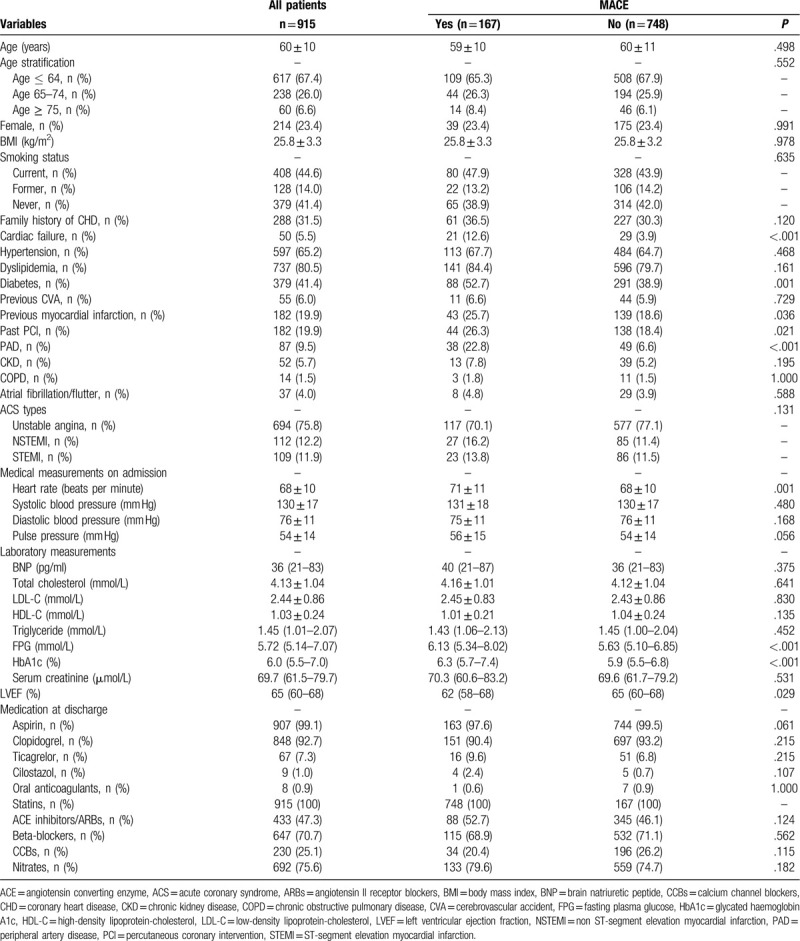
Baseline demographic and clinical characteristics of the overall population and after stratification by MACE at follow up.

**Table 2:**
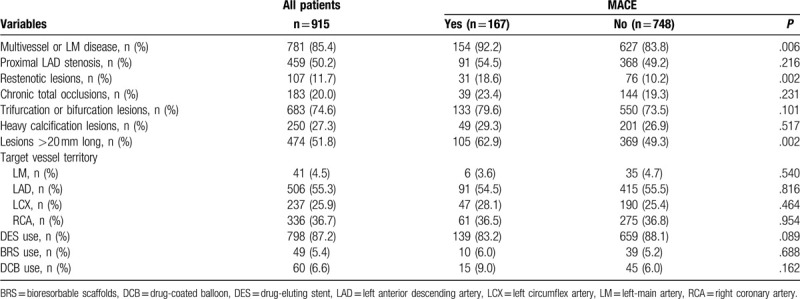
Angiographic findings and interventional characteristics of the overall population and after stratification by MACE at follow up.

**Figure 2:**
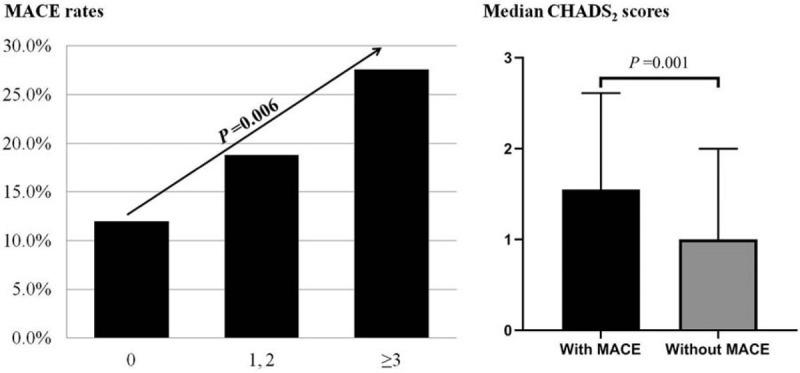
CHADS_2_ scores and rates of MACE. Left: Rates of MACE among low-, intermediate-, and high-CHADS_2_ score groups. Right: CHADS_2_ scores compared between patients with and without MACE. MACE = major adverse cardiovascular events.

**Figure 3:**
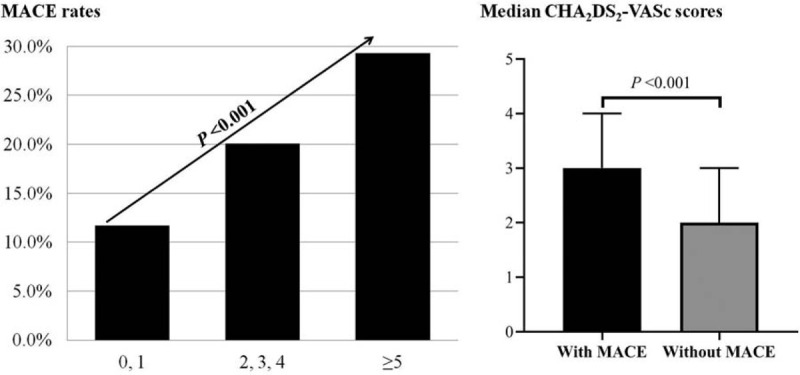
CHA_2_DS_2_-VASc scores and rates of MACE. Left: Rates of MACE among low-, intermediate-, and high-CHA_2_DS_2_-VASc score groups. Right: CHA_2_DS_2_-VASc scores compared between patients with and without MACE. MACE = major adverse cardiovascular events.

The rates of MACE among low-, intermediate-, and high-CHADS_2_ score groups were 12.0%, 18.8%, and 27.6%, respectively (*P* = .006; Fig. 2, left side). Patients with MACE had significantly higher CHADS_2_ scores than those without, 1.5 (1–2.5) vs 1 (0.5–2; *P* = .001; Fig. 2, right side). The rates of MACE among low-, intermediate- and high-CHA_2_DS_2_-VASc score groups were 11.7%, 20.1%, and 29.3%, respectively (*P* < .001; Fig. 3, left side). Patients with MACE had significantly higher CHA_2_DS_2_-VASc scores than those without, 3 (2–4) vs 2 (1–3; *P* < .001; Fig. 3, right side).

**Figure 4:**
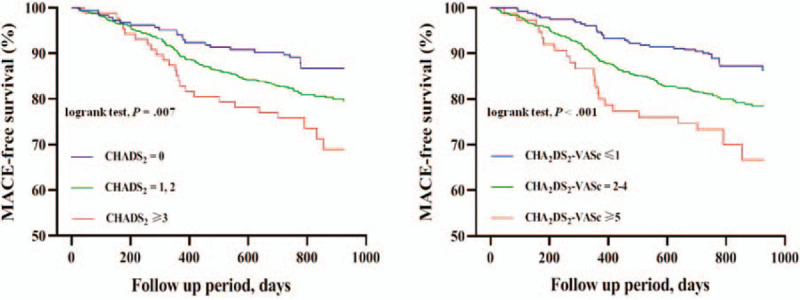
Kaplan–Meier unadjusted MACE-free survival rates stratified by CHADS_2_ and CHA_2_DS_2_-VASc score groups at follow-up. MACE = major adverse cardiovascular events.

Kaplan–Meier analysis showed that patients with higher CHADS_2_ scores had significantly reduced EFS from MACE (logrank test, *P* = .007; Fig. 4, left side). We saw a similar but larger difference in EFS from MACE when we stratified patients by CHA_2_DS_2_-VASc score (logrank test, *P* < .001; Fig. 4, right side). Rates of nonfatal stroke (logrank test, *P* < .001) were higher in proportion to CHADS_2_ score, but there was no significant difference in rates of overall death (logrank test, *P* = .098), CV death (logrank test, *P* = .082), nonfatal MI (logrank test, *P* = .148) or unplanned repeat revascularization (logrank test, *P* = .237) among the low-, intermediate-, and high-CHADS_2_ score groups. By contrast, incidence of nonfatal stroke (logrank test, P < .001) and unplanned repeat revascularization (logrank test, *P* = .027) were higher in proportion to CHA_2_DS_2_-VASc score, but there was no significant difference in rates of overall death (logrank test, *P* = .103), CV death (logrank test, *P* = .099) or nonfatal MI (logrank test, *P* = .288) among the low-, intermediate-, and high-CHA_2_DS_2_-VASc score groups.

Table 3 shows univariate and multivariate Cox proportional-hazard regression analyses (not including CHADS_2_ or CHA_2_DS_2_-VASc score) for MACE at follow up. In multivariate analysis, PAD, cardiac failure, heart rate on admission and serum levels of triglyceride were all independent predictors of MACE. Table 4 presents univariate and multivariate Cox proportional-hazard regression analyses of CHADS_2_ and CHA_2_DS_2_-VASc scores for MACE at follow-up. CHADS_2_ score was a good predictor of MACE in the univariate analysis; however, in the multivariate analysis it was no longer associated with increased risk of MACE. CHA_2_DS_2_-VASc score used as a continuous variable was independently predictive of MACE (HR, 1.15; 95% CI, 1.04–1.27; *P* = .007). When CHA_2_DS_2_-VASc score was used as an ordinal variable, multivariate analysis showed that compared with the low-score group used as a reference, the HRs of the intermediate- and high-score groups for predicting MACE were 1.52 (95% CI, 1.02–2.27; *P* = .042) and 1.96 (95% CI, 1.10–3.49; *P* = .022), respectively. Since CHA_2_DS_2_-VASc score reflects sex-specific stroke or systemic-embolism risk prediction, sex (male vs female) was subject to post-hoc subgroup analysis for MACE. When the analysis was stratified by sex, we found that a higher CHA_2_DS_2_-VASc score was significantly associated with increased risk of MACE in male patients (adjusted HR 1.20, 95% CI: 1.06–1.35; *P* = .004); however, the similar result did not occur in female patients (adjusted HR 1.10, 95% CI: 0.89–1.36; *P* = .373).

**Table 3:**
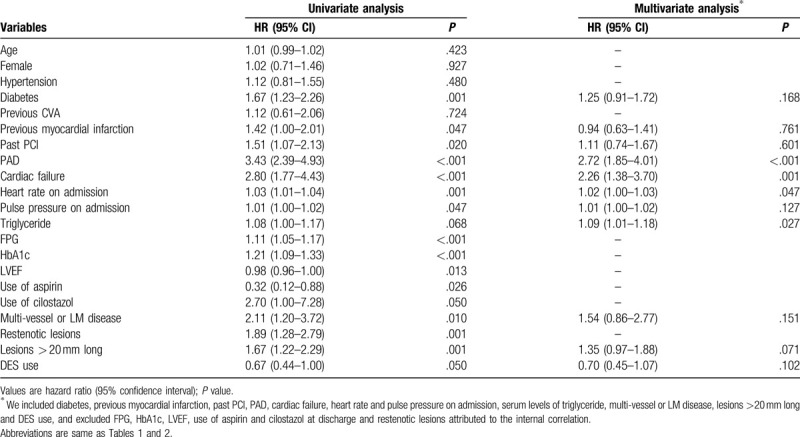
Cox proportional hazards regression analysis for MACE at follow up.

**Table 4:**
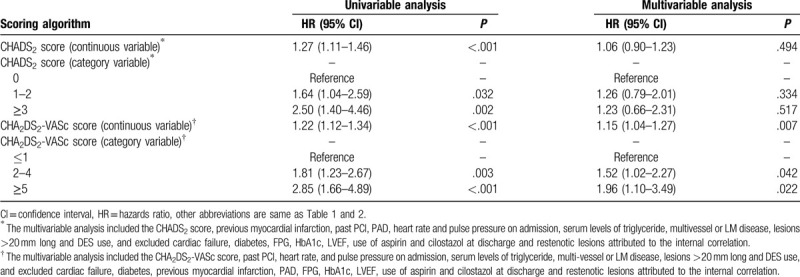
HRs for MACE predicted by the CHADS_2_/CHA_2_DS_2_-VASc scores.

To further evaluate the utility of CHA_2_DS_2_-VASc score for predicting MACE, we compared its discriminatory performance with those of the GRACE Score and SS II as continuous variables. In receiver operating characteristic (ROC) curve analyses, the CHA_2_DS_2_-VASc score had similar C-statistics for MACE as GRACE Score (C-statistics = 0.614; 95%CI, 0.564–0.663 vs C-statistics = 0.598; 95% CI, 0.550–0.645; *P* = .230) and SS II (C-statistics = 0.614; 95%CI, 0.564–0.663 vs C-statistics = 0.609; 95% CI, 0.566–0.652; *P* = .410).

## 4 Discussion

The CHADS_2_ and CHA_2_DS_2_-VASc scores were originally developed and validated as predictors of stroke or systemic-embolism risk and as stratification schemes for patients with nonvalvular AF to guide individualized antithrombotic therapy.^[3,4,28,29]^ Our study provides promise that CHA_2_DS_2_-VASc score can also play a role in predicting adverse CV outcomes. The main findings from this study can be summarized as follows: First, univariate analysis showed that CHADS_2_ score was a good predictor of MACE; however, it was no longer statistically significant after multivariate analysis. Second, CHA_2_DS_2_-VASc score independently and strongly predicted MACE in patients with ACS undergoing PCI, suggesting that this scoring system might have a potential role in early risk stratification of such patients. Finally, the discriminatory performance of the CHA_2_DS_2_-VASc score for MACE was comparable with those of GRACE Score and SS II.

The vast majority of CHA_2_DS_2_-VASc score components have been demonstrated to be independent predictors of adverse CV outcomes in ACS patients.^[2,30]^ This was one reason for the association between CHA_2_DS_2_-VASc score and adverse CV events in our study. CHA_2_DS_2_-VASc score is reported to be closely related to vascular endothelial dysfunction (VED) as assessed by brachial-flow–mediated dilation (bFMD) and brachial-ankle pulse wave velocity (baPWV).^[5]^ Adverse CV events such as MI^[31]^ and ischemic stroke^[32]^ have been demonstrated to be commonly pathophysiologically characterized by VED as reflected by reduced bFMD or increased baPWV. VED generally triggers the platelet adhesion and aggregation and the fibrin formation that play critical roles in systemic hypercoagulability.^[33]^ In addition, VED is reported to be significantly associated with CV risk factors such as cardiac failure,^[34]^ hypertension,^[35]^ and diabetes,^[36]^ which are components of the CHA_2_DS_2_-VASc score, and to be one of the key points of not only coronary-plaque vulnerability but also other CV complications such as vascular remodeling.^[37]^ The above-mentioned sequential associations might suggest a potential internal correlation between adverse CV events and CHA_2_DS_2_-VASc score.

The prognostic value of the CHA_2_DS_2_-VASc score has been validated in many different cohorts of patients with or without AF, but evidence for its application in ACS patients undergoing PCI is scarce. A study including 13,422 consecutive ACS patients from the Acute Coronary Syndrome Israeli Surveys (ACSIS) demonstrated that a higher CHA_2_DS_2_-VASc score was associated with increased in-hospital, 30-day and 1-year all-cause mortality, as well as with an increased incidence of 30-day combined endpoints of death, MI and unplanned revascularization.^[13]^ Similar findings were demonstrated in another study including 3184 ACS patients, which showed that a CHA_2_DS_2_-VASc score of ≥2 was associated with a higher rate of MI, stroke and death within 1 year of discharge, compared with a score of <2.^[7]^ In a recent prospective multicenter registry including 929 patients with AF who were referred for PCI, a high CHA_2_DS_2_-VASc score was found to significantly predict all-cause mortality, MI, repeat revascularization, stent thrombosis, TIA, stroke or other arterial thromboembolism.^[8]^ Interestingly, a study including 1330 non-AF patients undergoing PCI also demonstrated that CHA_2_DS_2_-VASc score could predict the combined endpoints of death, MI, destabilizing symptoms leading to hospitalization and nonfatal stroke after PCI.^[11]^ These reports suggest that CHA_2_DS_2_-VASc score has the potential to predict adverse CV events in ACS patients undergoing PCI. As our study demonstrated, a higher CHA_2_DS_2_-VASc score was associated with increased incidence of MACE. This finding was consistent with the results of several previous studies. An ACS subgroup (n = 7729) analysis of a recent study that included 12,785 patients with PCI showed that the CHA_2_DS_2_-VASc score predicted all-cause mortality and death or nonfatal MI in a significant (*P* < .001) and graded manner.^[38]^ Similarly, a study including 1729 consecutive patients with ACS undergoing PCI also demonstrated that CHA_2_DS_2_-VASc score was an independent predictor of adverse events including cardiac death, MI, stroke and any urgent coronary revascularization at long-term follow-up.^[16]^ Unfortunately, our study did not demonstrate an independent association between CHADS_2_ score and MACE. A potential explanation for CHADS_2_ score having no prognostic significance in our study, unlike CHA_2_DS_2_-VASc score, is that PAD, as the strongest predicator (HR, 2.01; 95% CI, 1.30–3.13) of MACE in the multivariate Cox proportional-hazard regression analysis (not including CHADS_2_ or CHA_2_DS_2_-VASc score), is not a component of in the CHADS_2_ score. Indeed, PAD is an established risk factor in ACS patients.^[19]^ In the post-hoc subgroup analysis based on sex, we found that a higher CHA_2_DS_2_-VASc score was an independent predictor of MACE in male patients, but no longer in female patients, which may be related to the following reason. In the present study, female patients had similar event rates to male patients (18.2% vs 18.3%), and thus sex was not independently associated with MACE in the overall patient cohort. However, the rates of MACE among low-, intermediate- and high- CHA_2_DS_2_-VASc score groups in female patients were all lower than those among the corresponding groups in male patients (5.9% vs 12.0%; 16.6% vs 21.4%; 28.3% vs 31.0%). In fact, female patients with ACS tend to have a higher CV risk than male patients because they may receive less medical attention and care. Therefore, we speculated that adjusted HR of 1.10 for MACE predicted by CHA_2_DS_2_-VASc score in female patients was not statistically significant due to limited sample size and follow-up time.

We demonstrated that the discriminatory performance of the CHA_2_DS_2_-VASc score for MACE was modest and acceptable. The study by Puurunen et al^[8]^ similarly found that the predictive performance of the CHA_2_DS_2_-VASc score was only modest (C-statistics = 0.57) for all-cause mortality and ischemic events in AF patients after PCI. In a recent retrospective cohort study including 1714 non-AF patients with coronary-artery disease (CAD) undergoing PCI from the SHINANO registry,^[9]^ the discriminatory performance of the CHA_2_DS_2_-VASc score for a composite endpoint including all-cause death, nonfatal MI and ischemic stroke was also shown to be modest (C-statistics = 0.64). In our study, we also demonstrated that the discriminatory performance of the CHA_2_DS_2_-VASc score was not inferior to that of the well-known GRACE Score. The GRACE Score is recommended by current guidelines to help clinicians determine post-discharge prognoses of ACS patients and might therefore be useful in guiding management strategy.^[39]^ The study by Capodanno et al^[11]^ revealed that the discriminatory performance of the GRACE Score (C-statistics = 0.66) was comparable with that of the CHA_2_DS_2_-VASc score (*P* = .15) for a composite ischemic endpoint including overall death, MI, destabilizing symptoms leading to hospitalization and nonfatal stroke in an ACS subgroup analysis of patients without AF undergoing PCI. Based on specific anatomical variables and several clinical comorbidities that were shown to affect mortality in the landmark all-comers SYNTAX trial, SS II was introduced to clinical practice for risk stratification in patients with complex CAD who have undergone revascularization. Recently, a study including 734 ACS patients undergoing PCI from the Special Programme University Medicine (SPUM) and COMFORTABLE AMI cohorts demonstrated that SS II was an independent predictor of death and ischemic events during 1-year follow-up and showed superiority in discriminating risk over conventional SS and GRACE Score for all-cause mortality.^[40]^ Our study is the first to assess the incremental prognostic value of the CHA_2_DS_2_-VASc score versus SS II in ACS patients undergoing PCI. C-statistics for SS II was similar to that for CHA_2_DS_2_-VASc score (0.609 vs 0.614, *P* = .410). Another study including 845 AF patients with coronary stenting reached a similar finding, showing discriminatory performance for adverse CV events (including death, stroke, acute MI, and target lesion revascularization) between CHA_2_DS_2_-VASc score and SS II (C-statistics = 0.54 vs 0.55).^[12]^ Compared with GRACE Score and SS II, CHA_2_DS_2_-VASc score has one great advantage: it provides a fast, simple and low-cost method for general clinicians as well as for cardiologists in risk assessment, requiring neither a calculator nor a computer. Therefore, CHA_2_DS_2_-VASc score may be a more practical scoring system for early risk stratification of patients with ACS undergoing PCI.

### 4.1 Limitations

A number of limitations should be noted in our study. First, follow-up information was obtained by telephone contact with patients or their relatives, even though medical records were always reviewed in case of ischemic events. Second, whether the findings from the present study including only Chinese patients can be extrapolated to other ethnic groups will require further studies. Third, all patients in our study had been treated with PCI; therefore, our results are not applicable to patients undergoing CABG or who were treated conservatively. Fourth, since we had excluded patients with prior CABG, renal dysfunction with CrCl < 15 mL/min or chronic dialysis, Killip class >II, NYHA class III/IV, or LVEF < 30%, our results may not be applicable to such patients. Fifth, at each follow-up we recorded medications the patients were taking, and found that medication adjustments according to patients’ conditions were frequent, especially 1 year after PCI. In fact, medication adjustments do have an impact on CV outcomes. Unfortunately, we did not include medication adherence in the analysis. Finally, CHADS_2_ and CHA_2_DS_2_-VASc scores do not take into consideration several angiographic variables and admission parameters such as cardiac biomarkers, ECG findings, or hemodynamic status. Nevertheless, differences in outcomes according to CHA_2_DS_2_-VASc score remained independent after multivariate Cox proportional-hazard regression analysis that included available angiographic and clinical characteristics.

## 5 Conclusions

CHA_2_DS_2_-VASc score, a simple and readily available score, could independently and strongly predict post-discharge outcomes in ACS patients undergoing PCI. Such findings need further independent confirmation in future large-scale studies. The potential role of CHA_2_DS_2_-VASc score in predicting MACE might offer important opportunities to optimize individualized treatment for risk-reduction management.

## Acknowledgments

We thank LetPub (www.letpub.com) for their linguistic assistance during the revision of this manuscript.

## Author contributions

Zhijian Wang and Yujie Zhou conceived and supervised the study; Xiaoteng Ma, Hua Shen and Jing Liang designed the study; Xiaoteng Ma, Qiaoyu Shao and Lisha Dong performed the study; Xiaoteng Ma, Sai Lv and Yujing Cheng analysed data; Xiaoteng Ma wrote the manuscript; Yujie Zhou made manuscript revisions. All authors reviewed the results and approved the final version of the manuscript.

## References

[1] Makki N Brennan TM Girotra S Acute coronary syndrome. J Intensive Care Med 2015;30:186–200. 24047692 10.1177/0885066613503294

[2] Abu-Assi E Lopez-Lopez A Gonzalez-Salvado V The risk of cardiovascular events after an acute coronary event remains high, especially during the first year, despite revascularization. Rev Esp Cardiol (Engl Ed) 2016;69:11–8. 26342640 10.1016/j.rec.2015.06.015

[3] Gage BF Waterman AD Shannon W Validation of clinical classification schemes for predicting stroke: results from the National Registry of Atrial Fibrillation. JAMA 2001;285:2864–70. 11401607 10.1001/jama.285.22.2864

[4] Lip GY Nieuwlaat R Pisters R Refining clinical risk stratification for predicting stroke and thromboembolism in atrial fibrillation using a novel risk factor-based approach: the euro heart survey on atrial fibrillation. Chest 2010;137:263–72. 19762550 10.1378/chest.09-1584

[5] Chan YH Yiu KH Lau KK The CHADS2 and CHA2DS2-VASc scores predict adverse vascular function, ischemic stroke and cardiovascular death in high-risk patients without atrial fibrillation: role of incorporating PR prolongation. Atherosclerosis 2014;237:504–13. 25463082 10.1016/j.atherosclerosis.2014.08.026

[6] Poci D Hartford M Karlsson T Role of the CHADS2 score in acute coronary syndromes: risk of subsequent death or stroke in patients with and without atrial fibrillation. Chest 2012;141:1431–40. 22016485 10.1378/chest.11-0435

[7] Chua SK Lo HM Chiu CZ Use of CHADS(2) and CHA(2)DS(2)-VASc scores to predict subsequent myocardial infarction, stroke, and death in patients with acute coronary syndrome: data from Taiwan acute coronary syndrome full spectrum registry. PLoS One 2014;9:e111167. 25343586 10.1371/journal.pone.0111167 PMC4208805

[8] Puurunen MK Kiviniemi T Schlitt A CHADS2, CHA2DS2-VASc and HAS-BLED as predictors of outcome in patients with atrial fibrillation undergoing percutaneous coronary intervention. Thromb Res 2014;133:560–6. 24461143 10.1016/j.thromres.2014.01.007

[9] Hioki H Miura T Miyashita Y Risk stratification using the CHA2DS2-VASc score in patients with coronary heart disease undergoing percutaneous coronary intervention; sub-analysis of SHINANO registry. Int J Cardiol Heart Vasc 2015;7:76–81. 28785649 10.1016/j.ijcha.2015.02.007 PMC5497243

[10] Melgaard L Gorst-Rasmussen A Lane DA Assessment of the CHA2DS2-VASc score in predicting ischemic stroke, thromboembolism, and death in patients with heart failure with and without atrial fibrillation. JAMA 2015;314:1030–8. 26318604 10.1001/jama.2015.10725

[11] Capodanno D Rossini R Musumeci G Predictive accuracy of CHA2DS2-VASc and HAS-BLED scores in patients without atrial fibrillation undergoing percutaneous coronary intervention and discharged on dual antiplatelet therapy. Int J Cardiol 2015;199:319–25. 26241637 10.1016/j.ijcard.2015.07.064

[12] Fauchier L Lecoq C Ancedy Y Evaluation of 5 prognostic scores for prediction of stroke, thromboembolic and coronary events, all-cause mortality, and major adverse cardiac events in patients with atrial fibrillation and coronary stenting. Am J Cardiol 2016;118:700–7. 27453515 10.1016/j.amjcard.2016.06.018

[13] Rozenbaum Z Elis A Shuvy M CHA2DS2-VASc score and clinical outcomes of patients with acute coronary syndrome. Eur J Intern Med 2016;36:57–61. 27707608 10.1016/j.ejim.2016.09.010

[14] Lu DY Huang CC Huang PH Usefulness of the CHADS2 score for prognostic stratification in patients with coronary artery disease having coronary artery bypass grafting. Am J Cardiol 2017;119:839–44. 28057218 10.1016/j.amjcard.2016.11.035

[15] Orvin K Levi A Landes U Usefulness of the CHA2DS2-VASc score to predict outcome in patients who underwent transcatheter aortic valve implantation. Am J Cardiol 2018;121:241–8. 29157734 10.1016/j.amjcard.2017.10.012

[16] Scudiero F Zocchi C De Vito E Relationship between CHA2DS2-VASc score, coronary artery disease severity, residual platelet reactivity and long-term clinical outcomes in patients with acute coronary syndrome. Int J Cardiol 2018;262:9–13. 29602582 10.1016/j.ijcard.2018.03.086

[17] Parodi G Scudiero F Citro R Risk stratification using the CHA2DS2-VASc score in Takotsubo syndrome: data from the Takotsubo Italian Network. J Am Heart Assoc 2017;6: e006065. 10.1161/JAHA.117.006065 PMC5634272 28912212

[18] Unal S Acar B Yayla C Importance and usage of the CHA2DS2-VASc score in predicting acute stent thrombosis. Coron Artery Dis 2016;27:478–82. 27187546 10.1097/MCA.0000000000000388

[19] Inohara T Pieper K Wojdyla DM Incidence, timing,;1; and type of first and recurrent ischemic events in patients with and without peripheral artery disease after an acute coronary syndrome. Am Heart J 2018;201:25–32. 29910052 10.1016/j.ahj.2018.03.013

[20] Mehilli J Presbitero P Coronary artery disease and acute coronary syndrome in women. Heart 2020;106:487–92. 31932287 10.1136/heartjnl-2019-315555

[21] Ma X Dong L Shao Q Predictive performance of aortic arch calcification for clinical outcomes in patients with acute coronary syndrome that undergo percutaneous coronary intervention: a prospective clinical study. Medicine (Baltimore) 2019;98:e18187. 31770274 10.1097/MD.0000000000018187 PMC6890324

[22] Amsterdam EA Wenger NK Brindis RG 2014 AHA/ACC guideline for the management of patients with non-ST-elevation acute coronary syndromes: a report of the American College of Cardiology/American Heart Association Task Force on Practice Guidelines. J Am Coll Cardiol 2014;64:e139–228. 25260718 10.1016/j.jacc.2014.09.017

[23] Levine GN Bates ER Blankenship JC 2015 ACC/AHA/SCAI focused update on primary percutaneous coronary intervention for patients with ST-elevation myocardial infarction: an update of the 2011 ACCF/AHA/SCAI guideline for percutaneous coronary intervention and the 2013 ACCF/AHA guideline for the management of ST-elevation myocardial infarction. J Am Coll Cardiol 2016;67:1235–50. 26498666 10.1016/j.jacc.2015.10.005

[24] Cockcroft DW Gault MH Prediction of creatinine clearance from serum creatinine. Nephron 1976;16:31–41. 1244564 10.1159/000180580

[25] Smith SC Jr Feldman TE Hirshfeld JW Jr ACC/AHA/SCAI 2005 guideline update for percutaneous coronary intervention: a report of the American College of Cardiology/American Heart Association Task Force on Practice Guidelines (ACC/AHA/SCAI Writing Committee to Update 2001 Guidelines for Percutaneous Coronary Intervention). Circulation 2006;113:e166–286. 16490830 10.1161/CIRCULATIONAHA.106.173220

[26] Lip GY Frison L Halperin JL Identifying patients at high risk for stroke despite anticoagulation: a comparison of contemporary stroke risk stratification schemes in an anticoagulated atrial fibrillation cohort. Stroke 2010;41:2731–8. 20966417 10.1161/STROKEAHA.110.590257

[27] Farooq V van Klaveren D Steyerberg EW Anatomical and clinical characteristics to guide decision making between coronary artery bypass surgery and percutaneous coronary intervention for individual patients: development and validation of SYNTAX score II. Lancet 2013;381:639–50. 23439103 10.1016/S0140-6736(13)60108-7

[28] Lip GY Tse HF Management of atrial fibrillation. Lancet 2007;370:604–18. 17707756 10.1016/S0140-6736(07)61300-2

[29] Lip GY Tse HF Lane DA Atrial fibrillation. Lancet 2012;379:648–61. 22166900 10.1016/S0140-6736(11)61514-6

[30] Pancholy SB Shantha GP Patel T Sex differences in short-term and long-term all-cause mortality among patients with ST-segment elevation myocardial infarction treated by primary percutaneous intervention: a meta-analysis. JAMA Intern Med 2014;174:1822–30. 25265319 10.1001/jamainternmed.2014.4762

[31] Kuvin JT Patel AR Sliney KA Peripheral vascular endothelial function testing as a noninvasive indicator of coronary artery disease. J Am Coll Cardiol 2001;38:1843–9. 11738283 10.1016/s0735-1097(01)01657-6

[32] Kim J Cha MJ Lee DH The association between cerebral atherosclerosis and arterial stiffness in acute ischemic stroke. Atherosclerosis 2011;219:887–91. 21974846 10.1016/j.atherosclerosis.2011.09.013

[33] Yau JW Teoh H Verma S Endothelial cell control of thrombosis. BMC Cardiovasc Disord 2015;15:130. 26481314 10.1186/s12872-015-0124-z PMC4617895

[34] Fujisue K Sugiyama S Matsuzawa Y Prognostic significance of peripheral microvascular endothelial dysfunction in heart failure with reduced left ventricular ejection fraction. Circ J 2015;79:2623–31. 26489455 10.1253/circj.CJ-15-0671

[35] Brandes RP Endothelial dysfunction and hypertension. Hypertension 2014;64:924–8. 25156167 10.1161/HYPERTENSIONAHA.114.03575

[36] McClung JA Naseer N Saleem M Circulating endothelial cells are elevated in patients with type 2 diabetes mellitus independently of HbA(1)c. Diabetologia 2005;48:345–50. 15660261 10.1007/s00125-004-1647-5

[37] Lavi S McConnell JP Rihal CS Local production of lipoprotein-associated phospholipase A2 and lysophosphatidylcholine in the coronary circulation: association with early coronary atherosclerosis and endothelial dysfunction in humans. Circulation 2007;115:2715–21. 17502572 10.1161/CIRCULATIONAHA.106.671420

[38] Orvin K Bental T Assali A Usefulness of the CHA2DS2-VASC score to predict adverse outcomes in patients having percutaneous coronary intervention. Am J Cardiol 2016;117:1433–8. 27001448 10.1016/j.amjcard.2016.02.010

[39] Tang EW Wong CK Herbison P Global Registry of Acute Coronary Events (GRACE) hospital discharge risk score accurately predicts long-term mortality post acute coronary syndrome. Am Heart J 2007;153:29–35. 17174633 10.1016/j.ahj.2006.10.004

[40] Obeid S Frangieh AH Raber L Prognostic value of SYNTAX score II in patients with acute coronary syndromes referred for invasive management: a subanalysis from the SPUM and COMFORTABLE AMI cohorts. Cardiol Res Pract 2018;2018:9762176. 30356345 10.1155/2018/9762176 PMC6176297

